# “Plasmonics” in free space: observation of giant wavevectors, vortices, and energy backflow in superoscillatory optical fields

**DOI:** 10.1038/s41377-018-0112-z

**Published:** 2019-01-03

**Authors:** Guanghui Yuan, Edward T. F. Rogers, Nikolay I. Zheludev

**Affiliations:** 1 Centre for Disruptive Photonic Technologies, The Photonic Institute, School of Physical and Mathematical Sciences, Nanyang Technological University, Singapore, 637371 Singapore; 2 Optoelectronics Research Centre and Centre for Photonic Metamaterials, University of Southampton, Highfield, Southampton, SO17 1BJ UK; 3 Institute for Life Sciences, University of Southampton, Highfield, Southampton, SO17 1BJ UK

**Keywords:** Nanophotonics and plasmonics, Metamaterials

## Abstract

Evanescent light can be localized at the nanoscale by resonant absorption in a plasmonic nanoparticle or taper or by transmission through a nanohole. However, a conventional lens cannot focus free-space light beyond half of the wavelength *λ*. Nevertheless, precisely tailored interference of multiple waves can form a hotspot in free space of an arbitrarily small size, which is known as superoscillation. Here, we report a new type of integrated metasurface interferometry that allows for the first time mapping of fields with a deep subwavelength resolution ~*λ*/100. The findings reveal that an electromagnetic field near the superoscillatory hotspot has many features similar to those found near resonant plasmonic nanoparticles or nanoholes: the hotspots are surrounded by nanoscale phase singularities and zones where the phase of the superoscillatory field changes more than tenfold faster than a free-propagating plane wave. Areas with high local wavevectors are pinned to phase vortices and zones of energy backflow (~*λ*/20 in size) that contribute to tightening of the main focal spot size beyond the Abbe–Rayleigh limit. Our observations reveal some analogy between plasmonic nanofocusing of evanescent waves and superoscillatory nanofocusing of free-space waves and prove the fundamental link between superoscillations and superfocusing, offering new opportunities for nanoscale metrology and imaging.

## Plasmonic-like electromagnetic field structures can exist in free space

A novel metamaterial-based technique can generate tailored optical fields for nanoscale optical applications. In plasmonics, highly-structured electromagnetic states can be generated by the coupling of light and free electrons in metals. This enables researchers to localize light in applications such as photovoltaics and is used to miniaturize optical devices. Until recently, plasmonic fields have been generated near the surfaces of metallic nanostructures, but now Guanghui Yuan and Nikolay Zheludev at Nanyang Technological University, Singapore, and Southampton University, UK have demonstrated that the similar field structure can be created in free space, through the interference of multiple waves forming a “superoscillatory” focused hotspot. The team developed a new form of interferometry to map the features of optical fields at resolutions orders of magnitude shorter than the light wavelength. They showed that the electromagnetic field near the superoscillatory hotspot had similar characteristics to those generated at plasmonic nanostructures.

## Introduction

In recent years, plasmonics—coupled electromagnetic states of light and free electrons in metals—has become the dominant research direction in photonics. The main advantage of plasmonics is that it gives access to large wavevectors of evanescent fields near metallic nanostructures, which facilitates miniaturization and enhanced light localization in numerous applications (including sensors, photovoltaics, light harvesting, data storage, spectroscopy, ultracompact electro-optical devices, and optical interconnects) and underpins the functionalities of advanced photonic materials and metamaterials engineered at the nanoscale. Moreover, evanescent plasmonic fields are often highly structured and contain phase singularities, vortices, and energy backflow zones. The main drawback of plasmonics is the resistive Joule losses in metals, which leads to a rapid dissipation of energy in the nanostructures. Considerable efforts have been devoted to searching for novel plasmonic materials with functionalities and losses improved beyond those offered by conventional plasmonic media, where the material characteristics can be better than those offered by conventional plasmonic metals, such as gold and silver.

The main purpose of this paper is to bring to the attention of the growing nanophotonics research community that the attractive features of evanescent plasmonic fields—such as high localization and extremely rapid variations of fields, giant wavevectors, phase singularities, nanoscale vortices, and energy backflows—can be constructed not only in the immediate vicinity of a metallic plasmonic nanostructure but also in free space, and therefore in the absence of Joule losses. Such extreme features can be generated in free space by the diffraction of light on purposely constructed masks. Our work falls into the rich and fertile domain of singular optics and is stimulated and informed by the pioneering works of Michael Berry and other researchers who theoretically predicted and observed complex field patterns in free-space optics, including vortices and knots^1–5^, largely without references to the plasmonic analogy.

However, our work goes further and explores one of the most practically important and fundamentally challenging questions of optics—how to focus light into a small spot—and exposes a previously unnoticed analogy between focusing by plasmonic nanostructures and superoscillatory focusing in free space.

In plasmonics, light evanescently confined near nanoparticles or nanostructures with a rich spatial spectrum can change very rapidly and possesses high frequencies in its spatial spectrum. For instance, a plasmonic wave propagating along the end of a metallic taper can be concentrated down to nanometer dimensions^6^. Both an opaque screen with a small hole (typically a few tens of nanometers in diameter) and a tapered optical fiber can localize light into the diameter of the exit hole, even if the light’s wavelength is much larger than the hole. Such nearly point-like sources of evanescent light are used in the high-resolution imaging technique known as scanning near-field optical microscopy (SNOM). It is also known that evanescent electromagnetic fields can form vortices of energy flow (represented by the Poynting vector) in the near-field of a screen^7^, in the interfacial region under total internal reflection^4^, near resonant plasmonic nanostructures^8,9^ or dielectric nanoparticles^10,11^. However, the evanescent component of the field does not propagate into the free space and decays rapidly away from the nanostructures.

However, can light be localized (focused) to a very small hotspot in free space that is far away from structured media? For a forward-propagating plane electromagnetic wave at frequency *ω* in free space, the projections of the wavevector |***k***_0_| = *ω/c* on any given direction *i* are band-limited to *k*_*i*_ ∈ [−NA × *ω*/*c*, NA × *ω*/*c*], where *c* is the speed of light and NA is the numerical aperture of the focusing system. This band-limit of light’s spatial spectrum is often understood to yield the “diffraction limit”: light cannot be structured smaller than a certain scale using interference (2*π*/|*k*_max_ − *k*_min_| = *λ*/2NA). The common wisdom here results in the Abbe–Rayleigh rule for focusing by a conventional lens, which claims that in free space no lens can concentrate light into a spot smaller than half its wavelength.

As a matter of fact, within a finite interval band-limited functions can locally oscillate much faster than their highest Fourier component. This is known as the phenomenon of superoscillation^12,13^. As explained by Michael Berry, superoscillations are possible because in the Wigner representation the local Fourier transform can have both positive and negative values, which causes subtle cancellations in the Fourier integration over the entire function^14^. Examples of superoscillatory functions are given in ref. ^15^. Applied to optics, the existence of superoscillations implies that if a number of waves with wavevectors bandlimited to [−*ω*/*c*, *ω*/*c*] interfere in free space, the local spatial spectrum could contain a high value wavevector with projections outside of the [−*ω*/*c*, *ω*/*c*] band. Here, we use the definition of the local wavevector as ***k***_local_ = ∇*φ*, where *φ* is phase of the electromagnetic field in the locality and ∇ is the gradient operator (more general definitions of a wavevector for a three-dimensional field can be found in ref. ^16^). In fact, there is no fundamental limit on how large the local wavevector can be. As a result, the free-space optical field created by the interference of several band-limited waves, such as by diffraction of a plane wave on a structured mask, can have deeply sub-wavelength spatial features. Such arbitrarily small foci of electromagnetic energy exist in free space and are far away from any boundaries with nanostructures or scattering objects, thus offering a very powerful opportunity for developing far-field, label-free, super-resolution, and non-algorithmic microscopies at harmless levels of intensity without impregnating them with luminescent materials, which gives a crucial advantage over other super-resolution imaging techniques, such as stimulated emission depletion (STED)^17^ and single-molecule localization methods (SMLM)^18,19^, which require labeling an object with luminescent materials and a high illumination intensity.

Superoscillations have been extensively studied in far-field super-resolution optical focusing and imaging^20–25^, signal processing^26^, electron wave confinement^27^, wavefunction localization of a single photon^28^, light trapping^29^, ultrashort pulse generation^30^, and optical speckles^31^, to name a few. In spite of these intense interests in the subject, no one has yet reported direct experimental evidence of large local wavevectors of superoscillatory optical fields in free space, which are predicted to be closely linked to superoscillations^13^. Observing these effects is much more complex than demonstrating sub-Abbe–Rayleigh intensity hotspots: it requires not only an efficient generator of superoscillatory optical fields but also the capability to recover the phase *φ* of the superoscillatory portion of the field, hence the local wavevectors ***k***_local_ = ∇*φ*.

**Fig. 1:**
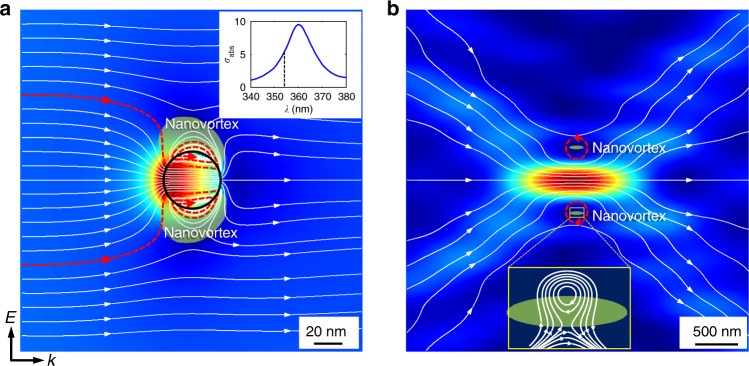
Focusing of light by a plasmonic nanoparticle and by a superoscillatory mask. **a** Plasmonic nanoparticle (evanescent fields): Poynting vector of light (white lines) near a plasmonic nanoparticle in resonance with an incident field following ref. [8]. The figure shows energy flow in the plane of polarization near a 40 nm-diameter silver nanoparticle illuminated at *λ* = 354 nm, where the black circle indicates the nanoparticle boundary. Notice the evanescent field nanovortices with energy backflow regions (shaded in green) at the edges of the nanoparticle. The inset shows the normalized absorption cross-section. **b** Superoscillatory focus (free-space fields): Poynting vector of light near a superoscillatory focus in free space. In both panels the white lines show the direction of the energy flow, and the red lines schematically highlight the energy vortices. The color maps indicate the absolute value of the time averaged Poynting vector (real part), with the intensity increasing from blue to red. Notice nanovortices with energy backflow regions (shaded in green) near the superoscillatory focus

In this work, we report the first direct experimental observations that “sub-diffraction” superoscillatory optical focus in free space is surrounded by zones where the magnitude of the local wavevector significantly exceeds |***k***_0_| = *ω*/*c*. Moreover, we show that the formation of superoscillatory foci breaking the Abbe–Rayleigh limit is linked to and facilitated by the formation of energy backflow zones in free space, which is far away from the structured mask that created the complex superoscillatory field. This brings us to an interesting theoretical prediction recently made by Berry^32^, who demonstrated that in a field created by the interference of multiple waves with positive wavevectors, the local phase gradient can sometimes be negative. Here, we show that energy backflow plays a crucial role in the formation of superoscillatory foci, which are pinned to phase singularities and energy nanovortices (see Fig. 1) even if they are far from any scattering objects.

In essence, our experimental observations reveal an intriguing similarity between the structures of evanescent fields focused by plasmonic nanostructures and those of free-space superoscillatory focus. In both cases, giant local wavevectors, phase singularities, energy vortices and zones of backflow are present, signifying and making the sub-wavelength localization of light possible (see Fig. 1).

## Results

### Nanoscale interferometry of superoscillatory fields

There are two main challenges in providing complete mapping of superoscillatory fields. First, since phase information can only be recovered in interferometric measurements, extreme stability of the interferometer is needed to obtain reliable data on fields with small spatial features and fast phase variations. Second, the superoscillatory fields are expected to have spatial features that are much smaller than the wavelength of light: a spatial resolution far better than that allowed by the Abbe–Rayleigh limit of a half-wavelength is required.

To meet these two challenges, we developed an original monolithic metasurface interferometry (MMI). In this technique, the superoscillatory field under investigation and reference wavefront needed for interferometry are created by the same planar metamaterial nanostructure, i.e., on the same monolithic platform, thus minimizing the issues of stability and alignment that are characteristic of free-space interferometers^33–35^.

Interference of the superoscillatory and reference fields creates a field distribution in free space that can be mapped with a linear detector array. The resolution of the detector array is determined by its pixel size and is insufficient for mapping. However, the field containing superoscillatory components and sub-wavelength features below the Abbe–Rayleigh diffraction limit is formed by the interference of free-space waves and can therefore be imaged with magnification by a lens with an NA exceeding that of the diffraction mask to collect all the wavevector components. We used a complementary metal-oxide–semiconductor (CMOS) camera array with a pixel size of 6.5 µm together with a high magnification optics (×500), thus achieving a spatial resolution of 13 nm. It will be shown below that the monolithic metasurface interferometric technique allows for the detection of superoscillatory field features that are below 2% of the wavelength in size.

**Fig. 2:**
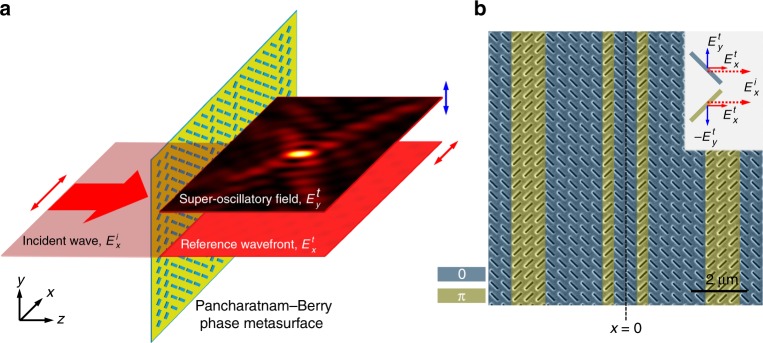
Monolithic metasurface interferometry with a superoscillatory Pancharatnam–Berry phase metasurface. **a** Principle of monolithic metasurface interferometry: a single metasurface creates the field distribution under investigation and the reference wave in orthogonal polarization. These two wavefronts are made to interfere on a polarization sensitive detector. **b** Scanning electron microscopy image of the 40 µm by 40 µm metasurface fabricated in a 100 nm-thickness gold film with focused ion beam milling (unit cell size 400 nm by 400 nm) that acts as a superoscillatory field generator and interferometric platform. Artificial colors indicate rows providing a binary 0/π phase shift in the transmitted light. The phase of light transmitted through the metasurface depends on the incident polarization and orientation of the slit, giving the opportunity to create a superoscillatory field for one of the transmitted polarizations and a plane wave for the orthogonal one, as explained in the text. A series of polarization-sensitive measurements of the intensity distribution of the diffracted light for different input polarizations allow for unambiguous recovery of the phase map of the superoscillatory field

The key component of the monolithic metasurface interferometer is a metamaterial mask (Pancharatnam–Berry phase metasurface^36–38^) that simultaneously creates the tight superoscillatory focus and the reference wavefront for the interferometry (see Fig. 2a). It contains rows of identical scattering subwavelength slits oriented at either +45° or −45° with respect to the *x*-axis and has translation symmetry in the *y* dimension, thus working similarly to a cylindrical lens focusing light into a line focus. The working principle of the mask is shown in Fig. 2b. The mask is designed to be polarization-sensitive and only creates a superoscillatory field in cross-polarization to the incident wave. The field co-polarized with the incident wave will propagate through the mask as a plane wave—with some attenuation. For example, when the metasurface is illuminated with *x*-polarized light ($E_x^{\mathrm {i}}$ on Fig. 2b), the phase of *x*-polarized transmitted light ($E_x^{\mathrm {t}}$) will be independent of the slit orientation: the *x*-polarized transmitted wave will remain a plane wave since the period is subwavelength and only the zero diffraction order is generated. However, the slits oriented at +45° and −45° to the *x*-axis will transmit *y*-polarized light with a π phase difference ($E_y^{\mathrm {t}}$ and $- E_y^{\mathrm {t}}$, see Fig. 2b). Therefore, the pattern of slits works as a binary phase grating for *y*-polarization. We define this arrangement as the TE configuration. Similarly, when illuminated with *y*-polarized light, the metasurface is a binary phase mask for the transmitted *x*-polarization (TM configuration).

Such a mask allows for a straightforward interferometry between the superoscillatory field and the reference plane wavefront that are mutually stable and inherently aligned by design. A 3D map of the intensity and phase can be recorded by measuring the intensity distributions at different distances from the mask using different input polarizations and polarization-sensitive detection.

Upon transmission through the metasurface, the *x*-polarized field suffers the same phase retardation regardless of the orientation of the slits and with the same intensity attenuation at all points due to the energy transfer into the cross-polarized field. Therefore, for the *x*-polarized field the metasurface is a homogeneous subwavelength grating of limited size (aperture). It will produce only a zero-order diffraction field, which does not depend on the state of the polarization of light incident on the metasurface. Although the *x*-polarized field shows some variations from the plane wave due to aperture diffraction at the edges of the metasurface, it is a good reference field for interferometry as it has a phase close to that of a plane wave (see Supplementary Information section A for details) and a well-defined, easy-to-measure intensity profile with no zeros.

In our experiment, the metasurface contains 100 rows of slits. The metasurface grating is designed with a particle swarm optimization algorithm to generate superoscillatory foci of the prescribed spot size, focal distance, field of view and depth of focus (see Materials and methods for the metasurface design details). Since the grating creates a superoscillatory focus for only one polarization while the transmitted light remains a plane wave for the other, the phase distributions in the TE and TM configurations $\varphi _{\mathrm {{TE,TM}}} = \arg \left( {E_{y,x}} \right)$ can be mapped by measuring the intensity distribution $I\left( {x,z} \right)$ of the interference pattern at a distance *z* from the mask for different polarizations of incident light (*x*, *y*, +45°, −45°, right and left circular) as follows:

1 $$\varphi _{\mathrm {{TE}}} = {\mathrm{atan}}\left( {\frac{{I_y^{\mathrm {{LCP}}} - I_y^{\mathrm {{RCP}}}}}{{I_y^{ + 45^\circ } - I_y^{ - 45^\circ }}}} \right) + k_0z$$

2 $$\varphi _{\mathrm {{TM}}} = {\mathrm{atan}}\left( {\frac{{I_x^{\mathrm {{RCP}}} - I_x^{\mathrm {{LCP}}}}}{{I_x^{ + 45^\circ } - I_x^{ - 45^\circ }}}} \right) + k_0z$$

Here, the second term on the right-hand sides of Eqs. (2) and (3) comes from the reference plane wave, and the superscripts and subscripts correspondingly denote polarization of the incident light and detection light. See Supplementary Information section B for the operating principle of the phase retrieval.

**Fig. 3:**
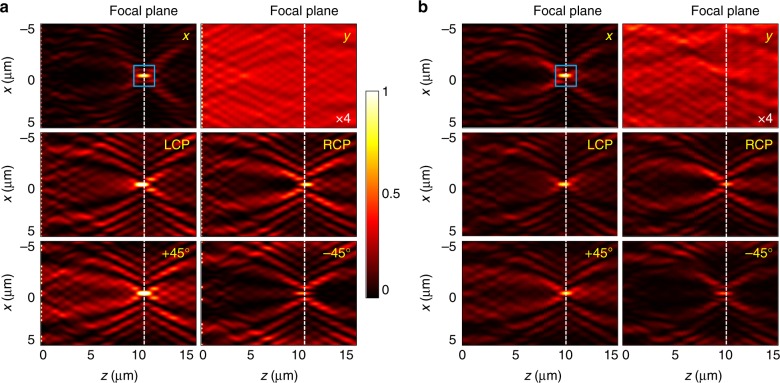
Superoscillatory field generated by the metasurface under different illumination conditions. **a** FDTD simulation and **b** experimental data. The figures show the *x*–*z* cross-section of the *y*-component intensity distribution in the interference pattern *I*_y_(*x*,*z*) for different incident polarizations (indicated in the corners of the maps—TE configuration). Note that the formation of the superoscillatory hotspot (highlighted in the blue box) located at a distance of 10 µm from the metasurface is best manifested for orthogonal incident polarization (*E*_*x*_). The quasi-uniform field map for the parallel incident polarization (*E*_*y*_) is multiplied by a factor of 4 and behaves like a reference plane wave for creating the interferogram with the signal superoscillatory field. The FDTD is computed and the experimentally measured maps show good agreement

The results of mapping the interference patterns $I\left( {x,z} \right)$ for the TE configuration can be found in Fig. 3, which shows performance of the metasurface evaluated by a finite-difference time-domain (FDTD) calculation. When the incident light at a wavelength of 800 nm is *x*-polarized, the *y*-polarized component of the diffracted wave contains a superoscillatory hotspot 10 µm away from the metasurface. The focal spot has a full-width at half-maximum (FWHM) of 0.42*λ*, which is well below the Abbe–Rayleigh diffraction limit *λ*/2NA = 0.56*λ* for a cylindrical lens with a numerical aperture corresponding to the experimental situation of NA = 0.89 (20 µm wide lens with a focal distance of 10 µm). Under *y*-polarized illumination, the *y*-polarized component of the diffracted wave is the reference field that we use for interferometry. For an infinitely long grating, it would show no structural features, while the minor variations in the transmission amplitude observed experimentally are due to the aperture effects. Its phase is uniform and therefore intensity variations do not affect the accuracy of the phase retrieval (see Supplementary Information section A). With circularly polarized incident waves, the diffraction patterns $I_y^{\mathrm {{RCP,LCP}}}\left( {x,z} \right)$ originate from the interference of the superoscillatory field and the reference wave with an initial phase difference of ±*π*/2 between them, depending on the handedness of the incident polarization. Similarly, for the incident linear polarization at ±45°, when we measure $I_{y}^{\pm 45^{\circ}}\left( {x,z} \right)$, the phase difference between the superoscillatory and reference fields becomes 0 and π, respectively. Now the phase map *φ*_TE_(*x*,*z*) of the superoscillatory field can be recovered from $I_y^{\mathrm {{RCP,LCP}}}\left( {x,z} \right)$ and $I_{y}^{\pm 45^{\circ}}\left( {x,z} \right)$ maps using formula (2).

In our experiment, we used an 800 nm wavelength diode laser as an optical source and mapped the interference pattern *I*(*x*, *z*) with a CMOS camera placed on a nanometric translation stage and equipped it with a ×500 magnification optical system. See Supplementary Information section C and Materials and methods for the detailed optical characterization setup. The corresponding experimental results for the intensity maps are given in Fig. 3b. Good agreement with the calculated maps is found in all field patterns. The co-polarization light shows intensity variations due to aperture diffraction on the mask edges. Some asymmetry in the pattern in Fig. 3b is due to imperfections in the incident wavefront. From a quantitative calculation, the mean square differences between a plane wave wavefront and the simulated and measured field maps were found to be 11.7% and 24.1%, respectively. Here, the superoscillatory hotspot (upper left panel in Fig. 3b) is also observed at *z* = 10 µm and its FWHM is measured to be 0.43*λ*, which is only about a 2% difference from the computed size of the hotspot.

### Four features of superoscillatory fields

**Fig. 4:**
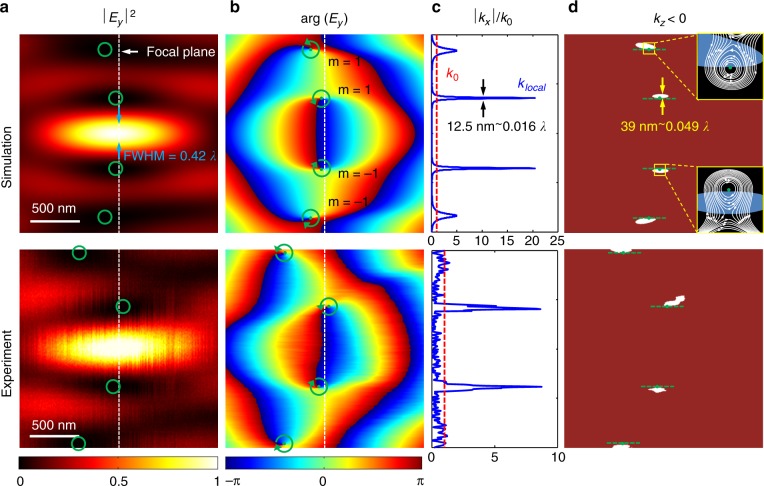
Four characteristic features of a superoscillatory field. (Top row) simulation; (Bottom row) experiment. **a** High localization of the field can be seen from the intensity map |*E*_*y*_|^2^ depicting focus with a FWHM of 0.42*λ*, smaller than that allowed by the Abbe–Rayleigh limit. The vertical dashed line indicates the plane of the focus. **b** Phase singularities with topological charge of *m* = ±1 (green dots) are seen on the phase maps arg(*E*_*y*_) in the low-intensity areas of the superoscillatory field. **c** Gigantic local wavevectors |*k*_*x*_|/*k*_0_ at the focal plane are calculated from the phase gradient in *x*-direction. Superoscillatory values of local wavevectors are highly localized in zones of order *λ*/100. **d** The energy backflow (retro-propagation) areas are painted white. They are substantially sub-wavelength in size (~*λ*/20 along *x*-direction) and correspond to negative values of *k*_*z*_ calculated from the phase gradient in the *z*-direction. Insets with the black background show a zoomed-in view of the time-averaged Poynting vectors near the phase singularity, and the backflow regions are shaded in blue. Dashed green lines indicate tangent to the retro-propagation areas at the point of their intersections with phase singularities

Figure 4a shows the computed and measured *x*–*z* cross-sections of the intensity map in the near vicinity of the superoscillatory focus, annotated by the blue box in Fig. 3. Here, we can see *the first characteristic feature of the superoscillatory optical field, namely, the high localization of the field*. The hotspot size in the *x*-section is smaller than that allowed by the Abbe–Rayleigh limit (because it does not take superoscillation into account). The focus is surrounded by fringes, similar to how the focal spot of a conventional lens of finite size is surrounded by the oscillating Airy pattern. However, here the fringes are more densely spaced than in the Airy pattern and much more extensive fringes are present. Indeed, superoscillatory hotspots are always surrounded by intense halos or fringes^15^. At the focal plane, the intensity of the first sidelobe is 17.6% (simulation) and 16% (experiment) of the peak intensity of the central hotspot.

The phase *φ*_TE_(*x*, *z*) of the electromagnetic field is rapidly changing near the superoscillatory focus, as shown in Fig. 4b. Here, one can observe a close match between the computed and experimentally measured phase maps that were retrieved from the intensity maps in Fig. 3b using formula (2). On the phase maps, one can clearly observe *the second characteristic feature of superoscillatory optical fields*: they are accompanied by phase singularities. At the low-intensity regions near the focus, one can see four phase singularities identified by green circles (Fig. 4b). When moving along the loop encircling the phase singular points, the phase changes by 2*π*. The two singularities in the upper part of the phase map have a topological charge of *m* = +1, while in the lower part they have topological charge of *m* = −1.

As *the third characteristic feature of a superoscillatory optical field*, we observed *gigantic local wavevectors* in the field maps with values far exceeding *k*_0_ = *ω*/*c*. First, we calculated the transverse wavevector *k*_x_ as the *x*-component of the gradient of the computed and measured the phase values. In the experiment, the pixilation of the phase mapping was 13 nm along the *x*-direction (determined by the effective pixel resolution of the detector) and 10 nm along the *z*-direction (scanning step size of the piezo stage). The normalized transverse local wavevector |*k*_x_|/*k*_0_ at *z* = 10 µm is given in Fig. 4c after data smoothing and interpolation with a step size of 5 nm. From there we see that the |*k*_*x*_| near the phase singularity is more than an order of magnitude higher than *k*_0_. Here, the computed and experimental data are in good agreement qualitatively. Although the data are somewhat smaller in the experiment, very large wave-numbers beyond the spectrum are still observed. Also note that the phase retrieval and wavevector mapping are robust to noise, see Supplementary Information section D on the noise sensitivity analysis.

The presence of *the fourth characteristic feature of a superoscillatory optical field*: the existence of *energy backflow* (*retro-propagation*) areas near the superoscillatory focus, which can be derived from the mapping of longitudinal wavevector *k*_*z*_. On the *x*–*z* maps we painted the areas of energy backflow as white zones in Fig. 4d. Indeed, we observed that *k*_*z*_ can have negative values near the phase singularities. Since the Poynting vector is parallel to the local wavevector in free-space, negative values of *k*_*z*_ mean energy back-flow (see insets presenting the computed time averaged Poynting vector maps $\left\langle S \right\rangle = {\Re} \left( {E \times H^ \ast /2} \right)$ near phase singularities). As can be clearly seen in the inset of Fig. 4d, phase singularities are pinned to the energy backflow regions, as predicted in ref. ^32^. Here, one can see that the incident energy flow is “trapped” and circulates without propagating in the forward direction (compare with energy flow near the plasmonic nanostructure, see Fig. 1). As predicted by Berry for a general case of interfering multiple waves^32^, “The boundaries of the retro-propagating regions include the phase singularities … and are tangent to the *z* direction at these points” (see green dashed lines on Fig. 4d. They are consistently directed along *z*, which confirms the accuracy of the experiment). Here, we also confirm another powerful observation from the same paper that “the regions of backflow are considerably smaller than the wavelength; this reflects the well-known fact that in the neighborhood of phase singularities wavefunctions can vary on sub-wavelength scales.” Indeed, backflow areas are only about *λ*/20 in size along the *x*-direction but are still satisfactorily resolved and their positions are accurately mapped on the computed locations. It is also noteworthy that due to the translation symmetry no energy backflow is observed in the plane perpendicular to the *x*–*z* plane. Note that similar optical vortices and energy backflow phenomena also exist in the longitudinally polarized field^39^.

The existence of phase singularities and energy backflow zones pinned to optical superoscillations gives a qualitative insight into the mechanism of focusing beyond the Abbe–Rayleigh limit. Two singularities that are close to the superoscillatory focus are located in the areas of diminishing intensity that define the boundary of the focus. At the superoscillatory focus, the backflow depletes the area where flow propagates in the forward direction, thus narrowing the focus beyond the conventional diffraction limit.

Similar results for the TM configuration are presented in Supplementary Information section E, where all the four characteristic features of superoscillatory fields, including the high localization of field, phase singularities, gigantic local wavevectors and energy backflow, are also experimentally observed. In addition, all these features can also be observed near resonant plasmonic nanoparticles. See an example in Supplementary Information section F.

## Discussion

Using new MMI, we comprehensively mapped a two-dimensional superoscillatory electromagnetic field generated by a Pancharatnam–Berry phase metasurface with an unprecedented resolution of ~*λ*/100. We have been able to experimentally prove and simultaneously observe, for the first time, four characteristic features of optical superoscillations: sub-Abbe–Rayleigh localization of light in the focus, phase singularities and nanovortices, gigantic local wavevectors and energy backflow (retro-propagation) in the immediate proximity of the focus. Our results will be useful for better understanding of superoscillatory focusing in wave optics^13,15,20,25^, electron waves^27^, and imaging applications^21–23^.

Our observations have identified some remarkable similarities between near-field plasmonic focusing by nanostructures and superoscillatory focusing in free space. Indeed, in both cases giant local wavevectors and phase singularities are observed surrounding the plasmonic and superoscillatory foci; energy vortices and zones of backflow are pinned to phase singularities and facilitate the sub-wavelength localization of light by removing the electromagnetic energy from the areas neighboring the foci (see Fig. 1). Although the degree of superoscillation in terms of the hotspot size here is only 75% of the Abbe–Rayleigh limit, a much higher degree of superoscillations is indeed possible.

We shall note that there is another important common feature of superoscillatory and near-field plasmonic focusing that does not directly follow from our observations, but rather represents basic scaling laws: the efficiency of focusing scales polynomially with the size of the focal spot. Indeed, a deeply sub-wavelength hole of diameter *σ* in an opaque screen can be used as a nanoscale light source in SNOM, for example. Only a small proportion of light illuminating the screen will pass through the hole. Here, the throughput efficiency scales as (*σ*/*λ*)^4^ + *o*(*σ*/*λ*)^4^ ^40^ A small absorbing dielectric or plasmonic nanoparticle of diameter *σ* will also “focus” light by harvesting energy of the illuminating wave (see Fig. 1a) with a scattering cross-section that scales as (*σ*/*λ*)^4^ ^41^. Similarly, in the regime of superoscillatory focusing in free space, only a small fraction of light can be focused in the hotspot. From the general theory of superoscillations^26^, it follows that the proportion of energy channeled into the superoscillatory region decreases polynomially *P*(*σ*/*λ*) with the size of the superoscillation.

Finally, we have shown that monolithic metasurface-based interferometry allows for robust generation of extremely small spatial features surrounding superoscillations, such as energy backflow zones and nanovortices by a single nanostructured metasurface. This finding offers interesting opportunities for metrology, such as measuring the lateral mutual displacement of two platforms when one platform contains the light source and the metasurface and the other an imaging light detector identifying the position of the sub-wavelength features projected on it by the metasurface.

## Materials and methods

### Metasurface design

The superoscillatory Pancharatnam–Berry phase metasurface is designed using the binary particle swarm optimization (BPSO) algorithm. The two-dimensional metasurface at the *x*–*y* plane is divided into *N* = 50 pairs of equally spaced rows of slits (Δ*x* = 400 nm) placed with mirror symmetry with respect to the *x* = 0 plane. Each row has slits oriented either at +45° or −45° with respect to the *x*-axis and provides phase delays of π and 0, respectively. The BPSO algorithm optimizes the light field distribution created by the metasurface near the focal plane when illuminated with a plane wave. The merit function to be optimized is defined as

3 $$I^{\mathrm {{tar}}}\left( {x,z} \right) = \left[ {{\mathrm{sinc}}\left( {ax} \right)} \right]^2{\mathrm{exp}}\left[ { - \left( {z - z_{\mathrm {f}}} \right)^2/b^2} \right]$$

where *z*_f_ is the desired focal length, *a* = 0.886/FWHM, $b = D{\mathrm{/2}}\sqrt {\ln 2}$, FWHM is the full-width at half-maximum of the hotspot size and *D* is the depth of focus.

### Sample preparation

The designed metasurface was fabricated by focused ion beam milling (FIB,Helios 650, Hillsboro, OR, USA) on a 100 nm-thick gold film deposited on a glass substrate using thermal evaporation (Oerlikon Univex 250, Cologne, Germany) with a deposition rate of 0.2 Å/s. The FIB writing voltage is 30 kV and the current is 7.7 pA.

### Optical characterization

The laser source is a diode laser with an emission wavelength of 800 nm (Toptica DLC DL pro 780, TOPTICA Photonics AG, Munich, Germany) and a linewidth of 100 kHz. A linear polarizer (P1) and a quarter waveplate (QWP) or half waveplate (HWP) are used to produce the desired incident polarization. The field distribution created by the metasurface was mapped with a high-resolution camera (Andor Neo sCMOS, Andor Technology Ltd., Belfast, UK; 2560 × 2160, pixel size 6.5 µm) and a high-magnification apochromatic planar objective corrected for field curvature (Nikon CFI LU Plan APO EPI (Nikon Instruments Inc., Melville, NY, USA) ×150, NA = 0.95) with a ×4 magnifier making distortion of the images practically negligible. The actual magnification factor is calibrated to be ~500, corresponding to an effective pixel size of 13 nm. Another polarizer (P2) is inserted into the optical path before the camera to select the desired detection polarization state (*E*_*y*_ in the TE case, *E*_*x*_ in the TM case). The field maps were obtained by *z*-scanning with a step size down to 10 nm using a piezo stage (PI E517, Physik Instrumente GmbH & Co., Karlsruhe, Germany).

## Supplementary information

Suppmentary Information
