# Supplementary material for: “Plasmonics” in free space: observation of giant wavevectors, vortices, and energy backflow in superoscillatory optical fields
Source: Light Sci Appl. 2019 Jan 3;8:2. doi: 10.1038/s41377-018-0112-z (PMC6318212; doi:10.1038/s41377-018-0112-z)
Supplement: Supplementary file 1 — Suppmentary Information [file 41377_2018_112_MOESM1_ESM.docx]

Supplementary Information for

**“Plasmonics” in free space: observation of giant wavevectors, vortices and energy backflow in superoscillatory optical fields**

Guanghui Yuan^1^, Edward T. F. Rogers^2,3^, and Nikolay I. Zheludev^1,2*^

^1^Centre for Disruptive Photonic Technologies, The Photonic Institute, School of Physical and Mathematical Sciences, Nanyang Technological University, 637371, Singapore

^2^Optoelectronics Research Centre and Centre for Photonic Metamaterials, University of Southampton, Highfield, Southampton SO17 1BJ, UK

^3^Institute for Life Sciences, University of Southampton, Highfield, Southampton SO17 1BJ, UK

*Corresponding author:* [^*^nzheludev@ntu.edu.sg](mailto:*nzheludev@ntu.edu.sg)

**Contents:**

1. Wavefront of reference wave
2. Operating principle of phase retrieval
3. Optical characterization setup
4. Sensitivity of phase retrieval to noise
5. TM configuration
6. Similarity between plasmonic and superoscillatory focusing
7. **Wavefront of reference wave**

To assess quality of the reference wave used in the interferometry, we simulated the field structure created by the metasurface using a finite-difference time-domain (FDTD) Maxwell equation solver (Lumerical, FDTD Solutions) for the TE configuration. A grid size of 10 nm, 10 nm, 5 nm is used in the *x*, *y*, *z* direction respectively. We applied the periodic boundary condition in the *y* direction and the perfect matched layer boundary condition in the *x* and *z* directions. The calculation results are shown in Fig. S1a. The reference wave $E_{x}^{x}$ shows a plane wavefront, although there is a slight intensity modulation due to the aperture effects and pixilation of the metasurface. Similar results are obtained for the TM configuration, confirming a flat wavefront for the reference wave $E_{y}^{y}$ (Fig. S1b).


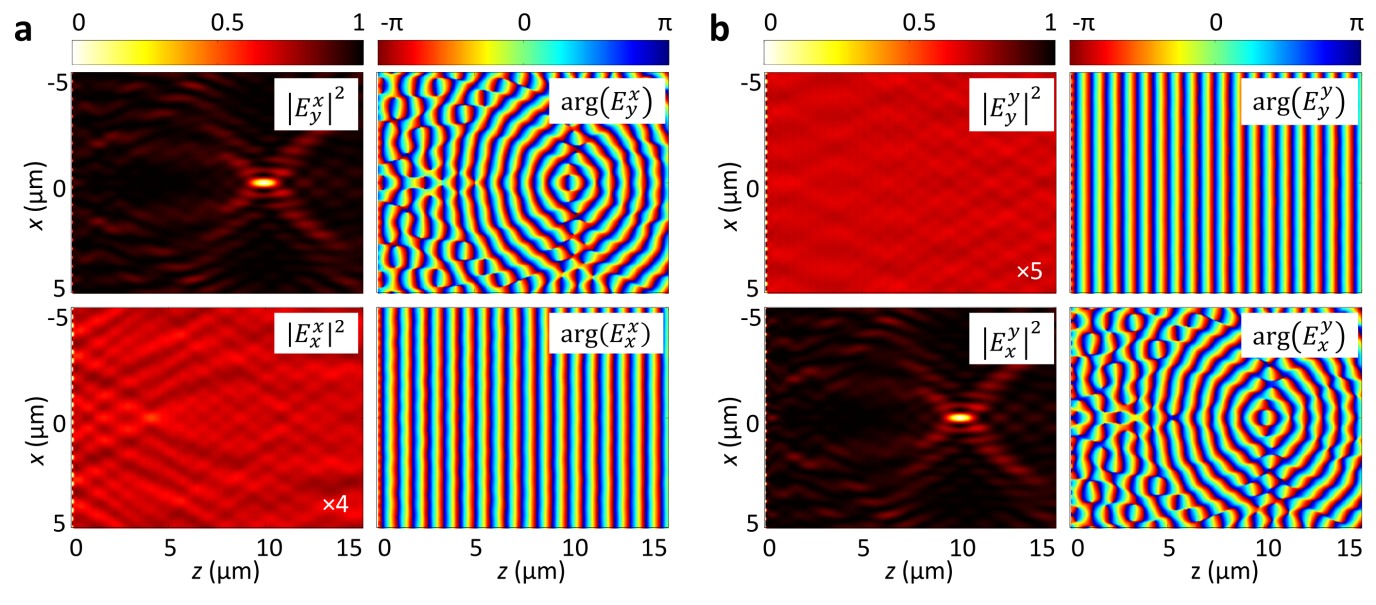


**Figure S1** FDTD simulated phase profiles of the superoscillatory field and reference wave. A plane wavefront could be observed in the reference waves although the intensity distributions have slight variations due to aperture effects. (**a**) TE configuration; (**b**) TM configuration.

1. **Operating principle of phase retrieval**

The designed metasurface produces a superoscillatory field in $E_{y}^{x}$ and a plane wave in $E_{x}^{x}$ under *x*-polarized excitation (see Fig. 2 in the main text), and a superoscillatory field in $E_{x}^{y}$ and a plane wave in $E_{y}^{y}$ under *y*-polarized excitation. Note that the superscripts and subscripts denote polarization of the incident field and detected field respectively. If illuminated by left-handed circularly polarized (‘*LCP*’) light, the transmitted field can be expressed as

$\left[\begin{matrix} E_{x}^{LCP} \\ E_{y}^{LCP} \end{matrix}\right]=\frac{1}{\sqrt{2}}\left[\begin{matrix} E_{x}^{x}+iE_{x}^{y} \\ E_{y}^{x}+iE_{y}^{y} \end{matrix}\right]$ (1)

And the corresponding intensity is given by

$\left[\begin{matrix} I_{x}^{LCP} \\ I_{y}^{LCP} \end{matrix}\right]=\left[\begin{matrix} \left(I_{x}^{x}+I_{x}^{y}\right)/2-\sqrt{I_{x}^{x}\cdot I_{x}^{y}}\sin\delta_{TM} \\ \left(I_{y}^{x}+I_{y}^{y}\right)/2+\sqrt{I_{y}^{x}\cdot I_{y}^{y}}\sin\delta_{TE} \end{matrix}\right]$ (2)

where is the phase difference between the superoscillatory field $E_{x}^{y}$ () and the plane wave $E_{x}^{x}$ (), while is the phase difference between the superoscillatory field $E_{y}^{x}$ () and the plane wave $E_{y}^{y}$ ().

Similarly, for right-handed circularly polarized light (‘*RCP*’) and ±45° linearly polarized light

$\left[\begin{matrix} I_{x}^{RCP} \\ I_{y}^{RCP} \end{matrix}\right]=\left[\begin{matrix} \left(I_{x}^{x}+I_{x}^{y}\right)/2+\sqrt{I_{x}^{x}\cdot I_{x}^{y}}\sin\delta_{TM} \\ \left(I_{y}^{x}+I_{y}^{y}\right)/2-\sqrt{I_{y}^{x}\cdot I_{y}^{y}}\sin\delta_{TE} \end{matrix}\right]$ (3)

$\left[\begin{matrix} I_{x}^{\pm45^{\circ}} \\ I_{y}^{\pm45^{\circ}} \end{matrix}\right]=\left[\begin{matrix} \left(I_{x}^{x}+I_{x}^{y}\right)/2\pm\sqrt{I_{x}^{x}\cdot I_{x}^{y}}\cos\delta_{TM} \\ \left(I_{y}^{x}+I_{y}^{y}\right)/2\pm\sqrt{I_{y}^{x}\cdot I_{y}^{y}}\cos\delta_{TE} \end{matrix}\right]$ (4)

From equations (2)-(4), we derive that:

$\text{tan}\left(\delta_{TM}\right)=\frac{I_{x}^{RCP}-I_{x}^{LCP}}{I_{x}^{+45^{\circ}}-I_{x}^{-45^{\circ}}}$ (5)

$\text{tan}\left(\delta_{TE}\right)=\frac{I_{y}^{LCP}-I_{y}^{RCP}}{I_{y}^{+45^{\circ}}-I_{y}^{-45^{\circ}}}$ (6)

And therefore,

(7)

(8)

Therefore, by measuring intensity maps ($I_{x}^{LCP}$, $I_{x}^{RCP}$, $I_{x}^{+45^{\circ}}$, $I_{x}^{-45^{\circ}}$) and ($I_{y}^{LCP}$, $I_{y}^{RCP}$, $I_{y}^{+45^{\circ}}$, $I_{y}^{-45^{\circ}},$), we are able to retrieve the phase of the superoscillatory field in TM and TE configuration respectively. The transverse and longitudinal local wavevectors can then be calculated from and .

It shall be noted that although we used straight slits metasurface here, other designs with large polarization-dependent diffraction efficiencies can also be used in monolithic metasurface interferometry.

1. **Optical characterization setup**


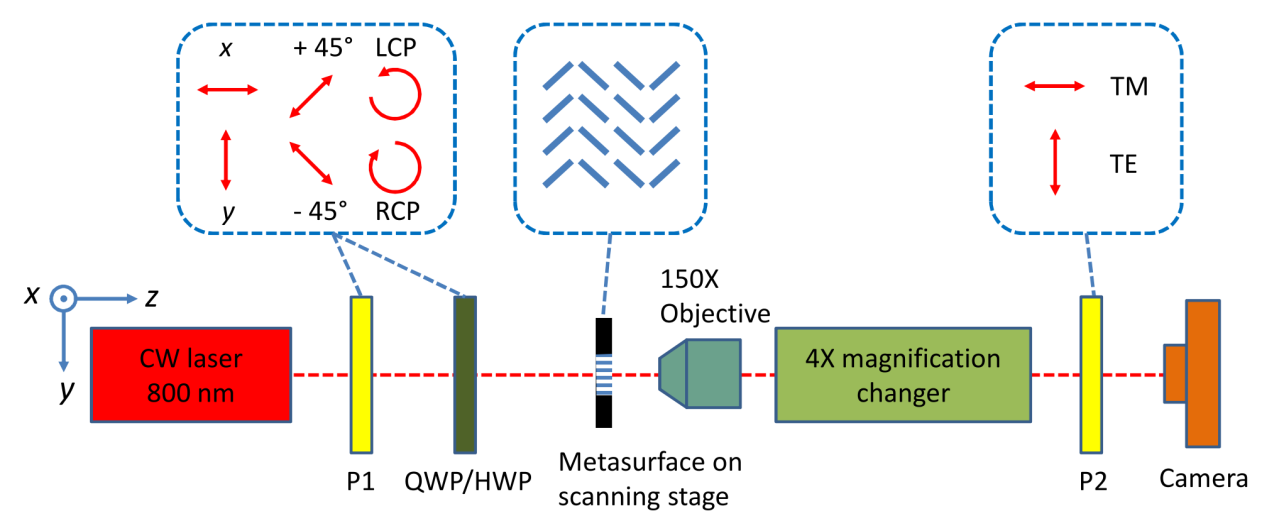


**Figure S2** Experimental setup for optical characterization. The superoscillatory field and reference plane wave are inherently aligned and coaxially propagating. P1, P2: linear polarizer; QWP: quarter waveplate; HWP: half waveplate. The top three panels show the *z*-view of the six incident polarizations (left) and two detection polarizations (right) used for intensity profile measurement and the schematic arrangement of the metasurface (middle).

1. **Sensitivity of phase retrieval to noise**

To address the question of stability of phase recovery from intensity maps using formula (2-3) of the main text, we simulated the impact of adding white noise to the phase retrieval process. The results are summarized in Fig. S3. Here the noise intensity level is defined as the ratio of the intensity fluctuation range to the maximum intensity of the focal spot at *z*=10 µm. In absence of noise, the phase and local wavevector are presented in Fig. S3a. Figure S3b shows the same maps at the noise level of 20%. All main features of the phase portrait are clearly seen, indicating excellent robustness of the phase recovery process to laser intensity fluctuations and noise in the CCD detector array.


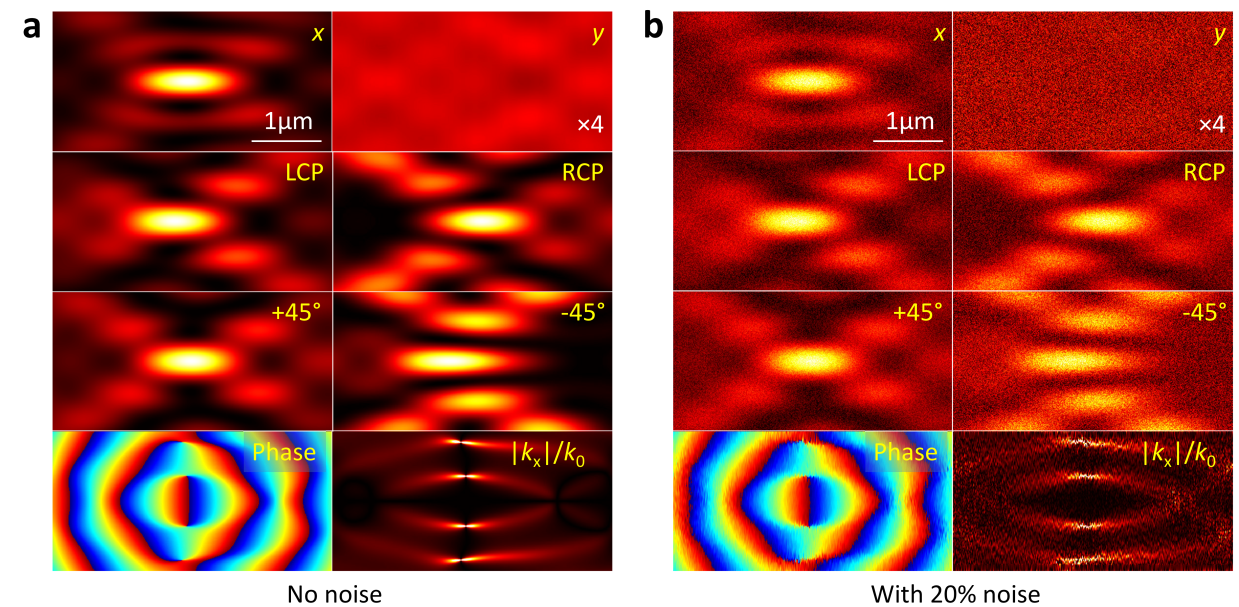


**Figure S3** Intensity, retrieved phase and local wavevectors distributions: (**a**) without noise; (**b**) with 20% noise.

1. **TM configuration**

In the main manuscript, we present results for the TE configuration. For completeness, we present equivalent field maps for the TM configuration in Figs. S4 & S5. The superoscillatory fields are created in $E_{x}$ under *y*-polarized excitation with calculated and experimentally achieved hotspot of 0.41𝜆 and 0.42𝜆 respectively. Four superoscillatory regions with are clearly observed in Fig. S5(c), and the energy backflow regions with $k_{z}<0$ highlighted in white are shown in Fig. S5(d).


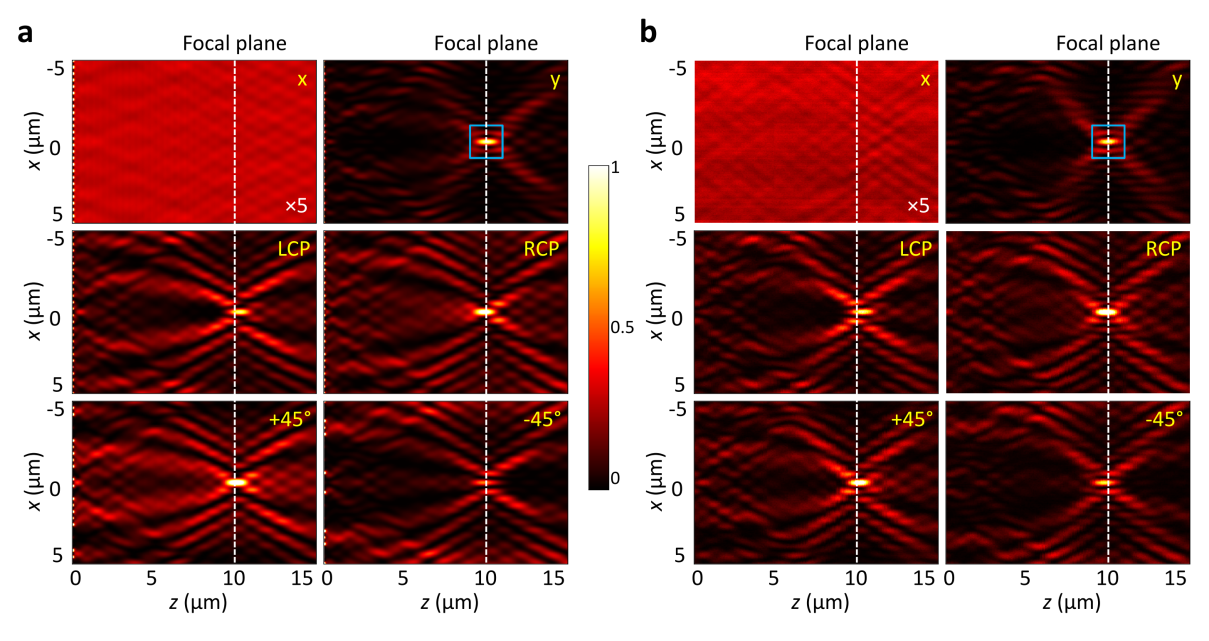


**Figure S4** Mapping of the field structures under TM configuration. (**a**) FDTD simulation; (**b**) Experimental data. The superoscillatory focus located at a propagation distance of 10 µm, as annotated by the blue box, is generated in *E*_x_ component under *y*-polarized excitation. A reference plane wave will be generated in *E*_x_ under *x*-polarized excitation, and its intensity is multiplied by a factor of five for clarity. Similar intensity measurement is taken under ‘*LCP*’, ‘*RCP*’, ‘+45°’ and ‘-45°’ excitation. FDTD computed and experimentally measured maps show good agreement.


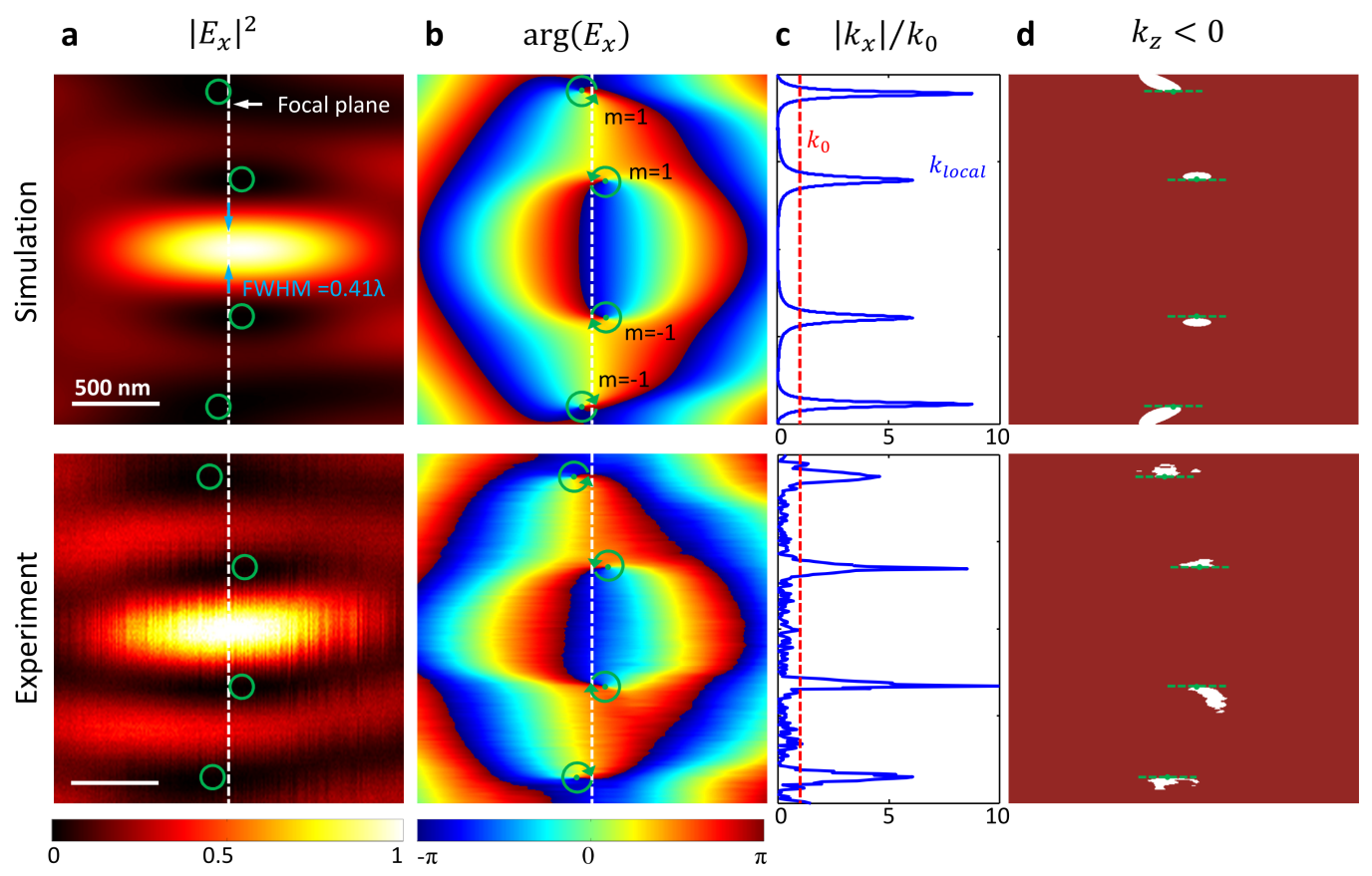


**Figure S5** Characteristic features of the superoscillatory field under TM configuration. (Top row) simulation; (Bottom row) experiment. (**a**) High localization of the field can be seen from the intensity map |*E*_x_|^2^ near the superoscillatory focus annotated in the blue box in Fig. S4; (**b**) Phase singularities with topological charge of *m* = +1 and -1 are seen on the phase maps arg(*E*_x_) in the low-intensity areas of the superoscillatory field highlighted by green circles; (**c**) Gigantic local wave-vectors |*k*_x_|/*k*_0_ at the focal plane are calculated from the phase gradient in *x*-direction; (**d**) The energy backflow areas with negative longitudinal wave-vector *k*_z_<0 are highlighted in white. Dashed green lines indicate tangent to the retro-propagation areas at the point of their intersections with phase singularities.

1. **Similarity between plasmonic and superoscillatory focusing**

To emphasize similarity between plasmonics and superoscillatory free-space fields, we compare the main four features of the super-oscillatory field described in the main text with the iconic case of the fields at plasmonic nanoparticle (see Fig. S6). The first characteristic feature of superoscillatory optical field, high localization of the field, is a well-known feature of plasmonic resonances that is well illustrated by Fig. S6a. The second characteristic feature of superoscillatory optical fields, phase singularities, can be seen from Fig. S6b. As in free-space superoscillatory fields, singularities are pinned to area of low intensity, see from the inset in Fig. S6a where the intensity near the interface shows a minimum. The third characteristic feature of superoscillatory optical field, gigantic local wavevectors, can also be seen in plasmonics, as shown in Fig. S6d. Moreover, as in free space superoscillatory fields, the large wavevectors |*k*_x_|>*k*_0_ (white areas on Fig. S6d) exist in the low-intensity regions (deep blue areas in Fig. S6a). The fourth characteristic feature of superoscillatory optical field: the energy backflow (retro-propagation) can also be seen in plasmonic field maps in Fig. S6c, which also exhibits another important feature of the free-space superoscillatory field observed in our work: the boundaries of the retro-propagating regions include the phase singularities (compare Fig. S6b and Fig. S6c).


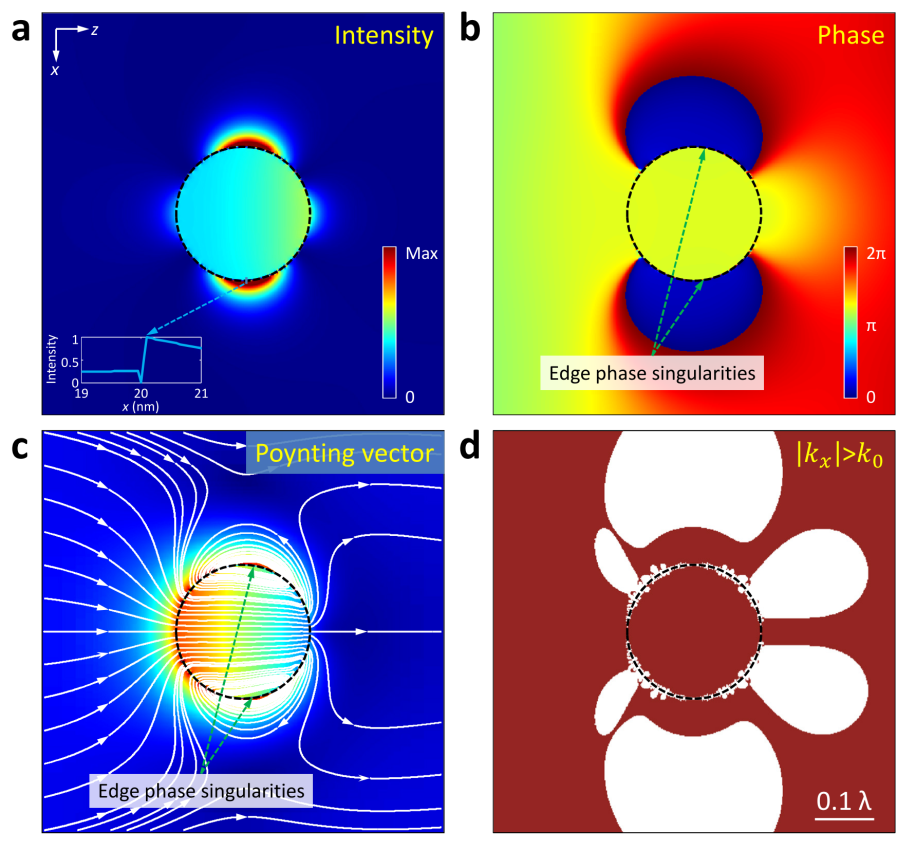


**Figure S6** High localization, phase singularities and energy backflow in plasmonic fields. (**a**) Intensity, (**b**) phase of the electric field near plasmonic resonance. The line field map across the boundary in (a) is shown in the inset where the low intensity region is clearly seen. (**c**) The Poynting vector map; (**d**) Large wavevector regions, areas with |*k*_x_|>*k*_0_ are coloured in white. All parameters are the same as for Fig. 1a in the main text. Black dashed circles show the boundary of the silver nanoparticle. The phase singularities are highlighted by the green dashed lines in (**b**) and (**c**).
